# Transcriptome Analysis of Dual FXR and GPBAR1 Agonism in Rodent Model of NASH Reveals Modulation of Lipid Droplets Formation

**DOI:** 10.3390/nu11051132

**Published:** 2019-05-21

**Authors:** Adriana Carino, Silvia Marchianò, Michele Biagioli, Chiara Fiorucci, Angela Zampella, Maria Chiara Monti, Elva Morretta, Martina Bordoni, Cristina Di Giorgio, Rosalinda Roselli, Patrizia Ricci, Eleonora Distrutti, Stefano Fiorucci

**Affiliations:** 1 Department of Surgical and Biomedical Sciences, University of Perugia, 06132 Perugia, Italy; adriana.carino@unipg.it (A.C.); silvia4as@hotmail.it (S.M.); michele.biagioli@unipg.it (M.B.); chiara.fiorucci125@gmail.com (C.F.); mbordoni92@gmail.com (M.B.); cristi.digiorgio@gmail.com (C.D.G.); patrizia.ricci@unipg.it (P.R.); 2 Department of Pharmacy, University of Naples Federico II, 80138 Naples, Italy; azampell@unina.it (A.Z.); rosellirosalinda@yahoo.it (R.R.); 3 Department of Pharmacy, University of Salerno, 84084 Fisciano, Italy; mcmonti@unisa.it (M.C.M.); emorretta@unisa.it (E.M.); 4 Azienda Ospedaliera di Perugia, Perugia 06156, Italy; eleonoradistrutti@katamail.com

**Keywords:** NASH, Liver disease, GPBAR1, FXR, BAR502

## Abstract

Non-alcoholic steatohepatitis (NASH) is a progressive, chronic, liver disease whose prevalence is growing worldwide. Despite several agents being under development for treating NASH, there are no drugs currently approved. The Farnesoid-x-receptor (FXR) and the G-protein coupled bile acid receptor 1 (GPBAR1), two bile acid activated receptors, have been investigated for their potential in treating NASH. Here we report that BAR502, a steroidal dual ligand for FXR/GPBAR1, attenuates development of clinical and liver histopathology features of NASH in mice fed a high fat diet (HFD) and fructose (F). By RNAseq analysis of liver transcriptome we found that BAR502 restores FXR signaling in the liver of mice feed HFD–F, and negatively regulates a cluster of genes including Srebf1 (Srepb1c) and its target genes—fatty acid synthase (Fasn) and Cell death-inducing DFF45-like effector (CIDE) genes, Cidea and Cidec—involved in lipid droplets formation and triglycerides storage in hepatocytes. Additionally, BAR502 increased the intestinal expression of Fgf15 and Glp1 and energy expenditure by white adipose tissues. Finally, exposure to BAR502 reshaped the intestinal microbiota by increasing the amount of *Bacteroidaceae*. In conclusion, we have shown that dual FXR/GPBAR1 agonism might have utility in treatment of NASH.

## 1. Introduction

Non-alcoholic Fatty Liver Disease (NAFLD) is a highly prevalent liver disorder worldwide [1]. NAFLD refers to a spectrum of conditions ranging from fatty liver disease, a relatively benign rarely progressive disease, to non-alcoholic steatohepatitis (NASH), a potentially progressive disease that carries a substantial risk of development of severe fibrosis, cirrhosis, and hepatocellular carcinoma [2]. While the pathogenesis of NASH is multifactorial, an excessive caloric intake is common among patients with NASH and, therefore, its prevalence is particularly elevated in obesity, type II diabetes, and metabolic syndrome [3,4]. Despite several agents being under development for treating NASH, as yet there are no drugs currently approved for its treatment [5]. 

Bile acids are signaling molecules that exert genomic and non-genomic effects by activating a group of cell surface and nuclear receptors collectively known as “bile acid activated receptors” (BARs) [6,7]. The Farnesoid-x-receptor (FXR) and the G-protein coupled receptor1 (GPBAR1 also known as TGR5), are the two best characterized receptors of the BARs family [8,9,10]. These receptors are mainly expressed in entero-hepatic tissues but also by cells of innate immunity, adipocytes, muscles, and vessels [6,7,8,9,10,11,12], and when activated by natural or synthetic ligands they regulate essential checkpoint in lipid, glucose, and protein metabolism. Specifically, FXR activation controls lipid metabolism and bile acid synthesis [13,14], and the pharmacological exploitation of FXR has led to the discovery of FXR agonists that have proven effective in reducing steatohepatitis in NAFLD and NASH patients [5,15]. In patients with NASH, the hepatic expression of FXR is downregulated, indicating a compromised FXR signaling in these patients. Accordingly, FXR expression levels are inversely correlated with disease severity, highlighting a potential mechanistic link between impaired FXR signaling and development of perturbed lipid metabolism [16]. Furthermore, FXR deficient mice spontaneously develop liver inflammation and fibrosis, due to impaired bile acid metabolism, while FXR agonism protects against the development of inflammation and fibrosis in various rodent models of liver injury [17,18]. Additionally, FXR activation regulates liver innate immunity [3,6,18], reduces lipogenesis, and promotes fatty acid β-oxidation [18], thereby protecting against hepatic steatosis. 

GPBAR1 is a cell surface receptor for secondary bile acids highly expressed in muscles, adipocytes, intestinal cells, and non-parenchymal liver cells, essentially liver resident macrophages and NKT cells [9,10,12]. In rodents, GPBAR1 is thought to increase energy expenditure [19], and its activation attenuates insulin resistance and increases basal metabolism by inducing mitochondrial respiration uncoupling and energy expenditure in white and brown adipose tissues [20]. Furthermore, GPBAR1 exerts potent anti-inflammatory effects [21], and its agonism corrects for endothelial dysfunction in rodent models of NASH [22,23]. Because of these diverse and only partially overlapping functions, the co-activation of FXR and GPBAR1 might hold promise in the treatment of metabolic disorders, including the NASH.

In 2002 we have reported the discovery of 6-ethyl-chenodeoxycholic acid [CDCA], then christened obeticholic acid (OCA), a semisynthetic derivative of CDCA [24], and shown that this agent effectively attenuates the development of liver steatosis in genetic models of NASH [25,26]. These findings have been confirmed later in clinical trials [15]. OCA is currently undergoing a phase 3 pre-registration trial for NASH [5]. The discovery that OCA causes severe side effects including pruritus (up to 70% of subjects with primary biliary cholangitis, PBC), and acute liver decompensation in cirrhotic patients [5], has raised concerns over the safety of FXR agonism in the treatment of severe liver diseases. However, since other non-steroidal FXR ligands have proven as effective as OCA in the treatment of NAFLD/NASH without the burden of the above mentioned side effects [5], this receptor should be considered a validated target in treating NASH. Following this line of research, we have recently reported the discovery of 6α-ethyl-3α, 7α-dihydroxy-24-nor-5β-cholan-23-ol (christened BAR502), a non-bile acid dual FXR and GPBAR1 agonist [27]. In a previous study, we have shown that BAR502 reverses features of steatohepatitis and fibrosis, and ameliorates lipid metabolism in a mouse-model of steatohepatitis [28,29]. 

In the present study, we have carried out a detailed analysis of the effect exerted by BAR502 investigating how the dual FXR/GPBAR1 agonist modulates the liver transcriptome by RNAseq analysis in mice fed a high fat diet (HFD) and fructose (F). Our results demonstrated that BAR502 functions as a negative regulator for a cluster of genes including Srebf1 (Srepb1c) and its target genes, fatty acid synthase (Fasn) and Cell death-inducing DFF45-like effector (CIDE) genes, Cidea and Cidec, involved in lipid droplets formation and triglycerides storage [30,31,32,33,34]. 

## 2. Material and Methods

### 2.1. Chemicals

Design and synthesis of BAR502 (6α-ethyl-3α, 7α-dihydroxy-24-nor-5β-cholan-23-ol) has been described previously [27,28]. BAR502 was dissolved daily in 1% methyl cellulose and administered by gavage (final volume 100 μL).

### 2.2. Animal Model

C57BL/6J male mice [29] were fed a high fat diet (HFD) containing 59 KJ% fat plus 1% cholesterol, without sugar (ssniff^®^ EF R/M acc. D12330 mod. 22,7 ME/Kg, Soest, Germany) and fructose (HFD–F) in drinking water (42 g/L) (45 mice), or normal chow diet (5 mice) for 12 weeks [20,21,23]. Food intake was estimated as the difference of weight between the offered and the remnant amount of food at 3-day intervals. The food was provided as pressed pellets and the residual spillage was not considered. After 10 days, HFD–F mice were randomized to receive HFD–F alone (10 mice) or in combination with BAR502, (20 mg/kg/day) (9 mice), by gavage for 11 weeks. Mice were housed under a controlled temperature (22 °C) and photoperiods (12:12-h light/dark cycle), and allowed unrestricted access to standard mouse chow and tap water. The experimental protocol was approved by the Animal Care and Use Committee of the University of Perugia and by the Italian Minister of Health and Istituto Superiore di Sanità (Italy) and was in agreement with the European guidelines for use of experimental animals (permission n. 583/2017-PR). The general health of the animals was monitored daily by the Veterinarian in the animal facility. At the day of sacrifice, fed mice were deeply anesthetized with a mixture of tiletamine hypochoride and zolazepam hypocloride/xylazine at a dose of 50/5 mg/Kg and sacrificed before 12 AM.

### 2.3. Thermal Images

BAT temperature was recorded through the study using a non-invasive technology. Briefly, mice were maintained at 25 °C and the thermal images were taken by a FLIR E6 thermal imaging camera (FLIR System, Wilsonville, OR, USA) and the surface temperature was quantified by the FLIR Tools [34]. 

### 2.4. Anthropometrical Determinations

The abdominal circumference (AC) (immediately anterior to the forefoot), body weight, and body length (nose-to-anus or nose–anus length), were measured in anaesthetized mice at time of sacrifice. The body weight and body length were used to calculate the Body mass index (BMI) (=body weight (g)/length^2^ (cm^2^)) and the Lee index (=cube root of body weight (g)/nose-to-anus length (cm)). 

### 2.5. Biochemical Analyses

AST, ALT, total- and HDL-cholesterol, and triglycerides were assayed using an automated clinical chemistry analyzer (Cobas, Roche). 

### 2.6. OGTT, Insulin Levels, and Bile Acids Assay

After 8 weeks of HFD–F mice were fasted overnight and orally administered glucose (1.5 g/kg body weight) for OGTT. The blood glucose concentrations were measured at 0, 15, 30, 60, 90, and 120 min after feeding or injection using a portable glucose meter (Accu-Check Go, Roche, Basilea, Switzerland) as described previously [28]. 

Gallbladder and fecal bile acid concentrations were measured by a liquid chromatography–tandem mass spectrometry (MS/MS), as described elsewhere [23,28,29]. 

### 2.7. Histopathology

For histological examination, portions of the right and left liver lobes were fixed in 10% formalin, embedded in paraffin, sectioned, and stained with Sirius red and/or Hematoxylin/Eosin (H&E), for morphometric analysis. NASH severity (steatosis, hepatocytes ballooning, lobular inflammation, and portal inflammation) was scored in H&E-stained cross sections using an adapted grading system of human NASH as described previously [20]. Hepatic fibrosis score was evaluated in Sirius red stained sections as described previously [17].

### 2.8. Mice Motor Activity

Mice motor activity was monitored using Panlab Infrared (IR) Actimeter (Panlab Harvard Apparatus, Barcelona, Spain) and analyzed with V2.7 ActiTrack software system [29]. Each animal motor activity was recorded for 10 min during the day every week. Data were shown as global motor activity i.e., sum of stereotypes and locomotion activity. Stereotyped movements during the analyzed interval, indicate the number of samples where the position of the mice is different from the position of the previous sample and equal to the position of the 2nd sample back in time. Locomotion activity indicates locomotion during the analyzed interval, i.e., distance walked in each sample where the position of the mice is different from the position of the previous sample and different of the position of the second sample back in time [23,29].

### 2.9. Quantitative Real-Time PCR analysis

RNA extracted from liver tissues was subjected to reverse transcription with RT^2^ First Strand Kit (Qiagen, Hilden, Germany). For Real Time PCR, 10 ng cDNA were amplified in a 20 μL solution containing 200 nM of each primer and 10 μL of RT^2^ SYBR Green ROX qPCR Mastermix (Qiagen). All reactions were performed in triplicate, and the thermal cycling conditions were as follows: 10 min at 95 °C, followed by 40 cycles of 95 °C for 10 s and 60 °C for 45 s in StepOnePlus instrument (Applied Biosystems, Foster City, CA, USA). The relative mRNA expression was calculated and expressed as 2^−(ΔΔCt)^. Expression of the respective gene was normalized against B2m and Gapdh mRNA as an internal control. The following primers for Real-Time PCR were used: mouse-Gapdh: ctgagtatgtcgtggagtctac and gttggtggtgcaggatgcattg; mouse-β2-microglobulin: ctttctggtgcttgtctcactg and ttcagcatttggatttcaatgt; mouse-Il-6: cttcacaagtcggaggctta and ttctgcaagtgcatcatcgt; mouse-Tnfα: ccaccacgctcttctgtcta and agggtctgggccatagaact; mouse-Il-1β: gctgaaagctctccacctca and aggccacaggtattttgtcg; mouse-F4/80: tgcaaaaggatcctcttcaagtg and actggggcacttttgttctca; mouse-Srebf1: gatcaaagaggagccagtgc and tagatggtggctgctgagtg; mouse-Fasn: tcaagatgaaggtggcagaggtgct and ttgagcagtgccgggattcgg; mouse-Bsep: gatgcttcccaagttcaagg and taaagaggaaggcgatgagc; mouse-Cyp7a1: aggcatttggacacagaagc and tgcatcatggcttcagagag; mouse-Cyp8b1: ttggccccatcattaagaac and ctggttgagttcctccaagc; mouse-Shp: acgatcctcttcaacccaga and agggctccaagacttcacac; mouse-Fgf21: cctgggtgtcaaagcctcta and ctccagcagcagttctctga; mouse-Fgf15: agacgattgccatcaaggac and ttcctccgagtagcgaatca; mouse-Glp1: ccccagatcattcccagctt and cgggagtccaggtatttgct; mouse-Prdm16: acggaagagcgtgagtacaaat and cgtgaacaccttgacacagttt; mouse-Ucp1: ctcactcaggattgtgcctctac and tctgaccttcacgacctctgta; mouse-Pparγ: gccagtttcgatccgtagaa and aatccttggccctctgagat; mouse-Pgc1α: cttagcactcagaaccatgcag and aatgctcttcgctttattgctc.

### 2.10. AmpliSeq Transcriptome

High-quality RNA was extracted from mice livers, using the PureLink™ RNA Mini Kit, according to the manufacturer’s instructions [30]. RNA quality and quantity were assessed with the Qubit^®^ RNA HS Assay Kit and a Qubit 3.0 fluorometer (Invitrogen, Carlsbad, CA, USA) followed by agarose gel electrophoresis. Libraries were generated using the Ion AmpliSeq™ Transcriptome Mouse Gene Expression Core Panel and Chef-Ready Kit (Thermo Scientific™, Waltham, MA, USA), (Comprehensive evaluation of AmpliSeq transcriptome, a whole transcriptome RNA sequencing methodology). Briefly, 10 ng of RNA was reverse transcribed with SuperScript™ Vilo™ cDNA Synthesis Kit before library preparation on the Ion Chef™ instrument. The resulting cDNA was amplified to prepare barcoded libraries using the Ion Code™ PCR Plate, and the Ion AmpliSeq™ Transcriptome Mouse Gene Expression Core Panel, Chef-Ready Kit, according to the manufacturer’s instructions. Barcoded libraries were combined to a final concentration of 100 pM, and used to prepare Template-Positive Ion Sphere™ Particles to load on Ion 540™ Chips, using the Ion 540™ Kit-Chef. Sequencing was performed on an Ion S5™ Sequencer with Torrent Suite™ Software v6. The analyses were performed with a range of fold <−2 and >+2, using Transcriptome Analysis Console Software (version 4.0.1), certified for AmpliSeq analysis (Thermo-Fisher, Waltham, MA, USA). 

### 2.11. Metagenomics

*DNA extraction.* The microbial DNA was purified from mouse stool samples, using the PureLink Microbiome DNA Purification Kit (Thermo Scientific™, Waltham, MA, USA), according to the manufacturer’s instructions. Briefly, approximately 100 mg of mouse stool was weighed and transferred to the Bead Tube and mixed thoroughly with 700 µL of S1-Lysis Buffer and 100 µL of S2-Lysis Enhancer to create a homogeneous sample and incubated at 65° 10 min. The Bead Tubes were homogenized for 10 min at maximum speed on the horizontal vortex mixer, then centrifuged at 14,000× *g* for 5 min and 400 µL of supernatant was transferred to a clean micro-centrifuge tube and vortexed immediately with 250 µL of S3-Cleanup Buffer. After 2 min of centrifugation, 500 µL of supernatant was transferred in a new Eppendorf and mixed with 900 µL of S4-Binding Buffer. Then 700 µL of sample mixture was loaded onto a spin column-tube and centrifuged at 14,000× *g* for 1 min (2X). The spin-column was then washed with 500 µL of S5-Wash Buffer and the flow-through was discarded. Finally the spin-column was placed in a clean tube, and the purified DNA was eluted with 100 µL of S6-Elution Buffer. The isolated DNA was quantified with a Qubit dsDNA HS Assay Kit on Qubit 3.0 Fluorometer (Thermo Scientific™, Waltham, MA, USA) according to the manufacturer’s instructions and then stored at −20 °C [35,36].

*16S rRNA sequencing.* Sequencing was performed using Ion 16S Metagenomics Kit (Thermo Scientific™, Waltham, MA, USA) on the Ion Torrent S5 platform (Thermo Scientific™, Waltham, MA, USA). Briefly, 3ng of DNA was subjected to amplification of 16S rRNA libraries using two primer pools to amplify seven hypervariable regions of bacterial 16S rRNA. Primers were partially digested and barcoded adapters (Ion Xpress Barcode Adapters 1-16 Kit) ligated to the amplicons, using the Ion Plus Fragment Library Kit (Thermo Scientific™, Waltham, MA, USA), purified using the Agencourt AMPure XP beads (Beckman Coulter, Brea, CA, USA) according to the manufacturer’s protocol, and stored at −20 °C until further processing. The concentration of each 16S library was determined by qPCR using the Ion Library Quantitation Kit and a Qubit 3.0 fluorometer (Thermo Scientific™, Waltham, MA, USA). The library was diluted to ~100 pM prior to template preparation. Template preparation of the barcoded libraries was performed using the Ion Chef and the Ion S5 System (Thermo Scientific™, Waltham, MA, USA). A maximum of 16 barcoded 16S samples were sequenced on an Ion 520 chip (Thermo Scientific™, Waltham, MA, USA) using the Ion 510 & Ion 520 & Ion 530 Kit - Chef (Thermo Scientific™, Waltham, MA, USA) according to the manufacturer’s instructions [35,36]. 

*Metagenomics Analysis.* Automated analysis, annotation, and taxonomical assignment were generated using Ion Reporter Software—Metagenomics Workflow (Ion Reporter 5.10.2.0 Thermo Scientific™, Waltham, MA, USA). The Ion Reporter Software enables the rapid identification (at genus or species level) of microbes present each sample, using both curated Greengenes and premium curated MicroSEQ ID 16S rRNA reference databases. The Ion Reporter metagenomics workflow also provides primer information, classification information, percent ID, and mapping information [35,36]. Data visualization and statistical analyses of taxonomy were performed using Krona and QIIME™ analysis softwares (http://qiime.org/), and related packages were used for diversity and correlation analyses. Principal coordinates analysis (PCoA) was conducted with identified reads/OTUs using classical multidimensional scaling (Bray-Curtis) to analyze distribution of dissimilarities and analysis of variance using abundance data.

### 2.12. Statistical Analysis

All of the data are shown as the means ± SEM. Difference among groups was estimated using one-way ANOVA followed by Tukey’s post hoc test, or by the T-test analysis, or by two-way ANOVA followed by Bonferroni’s post hoc test when appropriated (GraphPad Prism 5.0 software, San Diego, CA, USA). Significance was set up at *p* < 0.05.

## 3. Results

### 3.1. Effect of BAR502 on Body Weight and Biochemical Features of NASH

**Figure 1:**
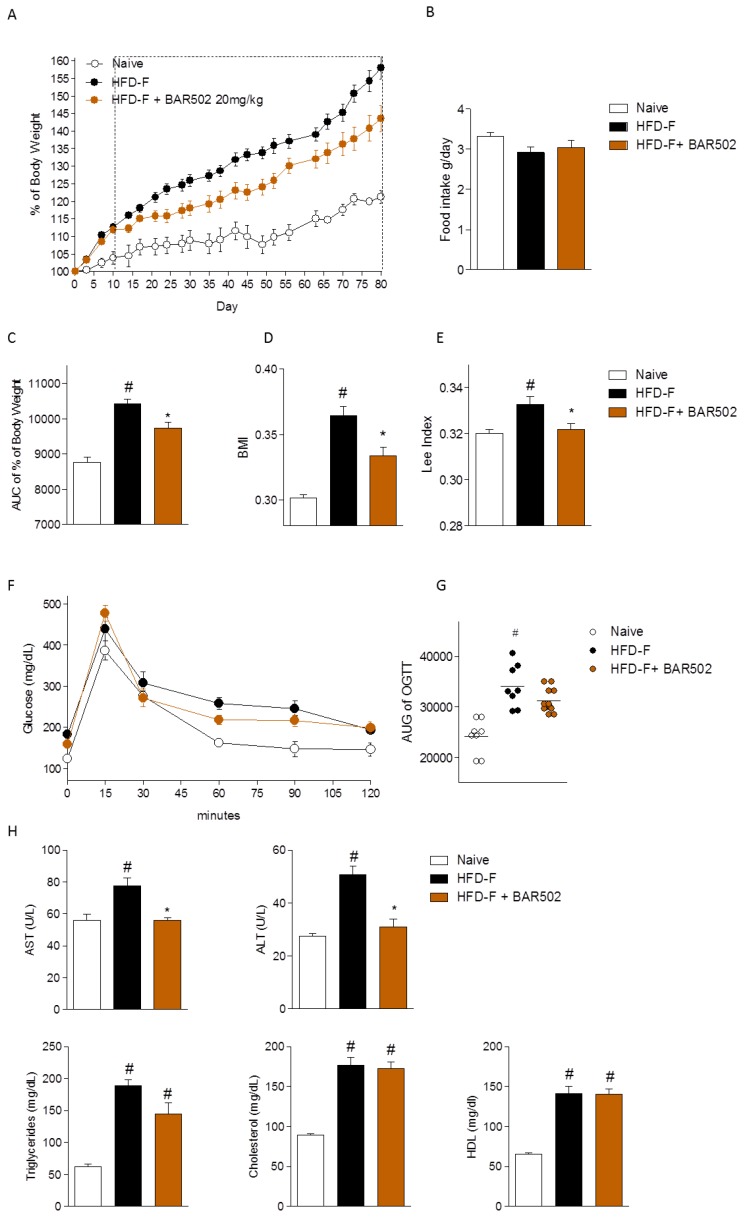
BAR502 reduces body weight gain, ameliorated insulin resistance and biochemical profile in mice fed a high fat diet and fructose (HFD–F). BAR502, 20 mg/kg/day, was administered by gavage in C57BL/6J male mice starting on day 10 of HFD and fructose for an additional 11 weeks. The data shown are: (**A**) % of body weight; (**B**) Food intake (g/day); (**C**) areas under curve (AUCs) of body weight expressed in arbitrary units; Anthropometrical parameters (**D**) body mass index (BMI) and (**E**) Lee Index, measured at the end of the study; (**F**) Glucose plasma levels response to oral glucose tolerance test (OGTT) and (**G**) AUCs of glucose plasma levels expressed in arbitrary units; (H) Plasma levels of aspartate transaminase (AST), alanine transaminase (ALT), Triglycerides, Cholesterol, and HDL and measured at the end of the study. The data shown are mean ± SE of 7–10 mice/group. In each panel # denotes *p* < 0.05 versus Naïve mice, * denotes *p* < 0.05 versus HFD–F mice.

We have first investigated whether treating mice with BAR502 rescues HFD–F mice from NASH-like features. As shown in Figure 1, exposure of mice to a high caloric diet increased body weight (≈35%) over naïve mice feed a normal chow diet and BMI and Lee Index. These two later indexes were measured at the end of the study (Figure 1A–E). Treating mice with BAR502 significantly reduced the body weight gain (AUC % of body weight) as well as changes in anthropometrical indexes caused by feeding mice a HFD–F (Figure 1C–E). 

After eight weeks of feeding a HFD–F, mice developed insulin resistance as shown by results of OGTT (Figure 1F). Treating mice with BAR502 effectively reversed this pattern and reduced the AUC of OGTT (Figure 1F,G, # *p* < 0.05 versus naïve mice, * *p* < 0.05 versus HFD–F mice). At the time of sacrifice, in comparison to naïve mice, mice fed a HFD–F alone, had increased plasma levels of AST, ALT, triglycerides, and cholesterol (Total and HDL) (Figure 1H, # *p* < 0.05 versus naïve mice). BAR502 effectively reduced AST, ALT, triglycerides but had no effect on cholesterol and HDL cholesterol (Figure 1H, # *p* < 0.05 versus naïve mice, * *p* < 0.05 versus HFD–F mice).

### 3.2. Effects of BAR502 on Liver Damage

**Figure 2:**
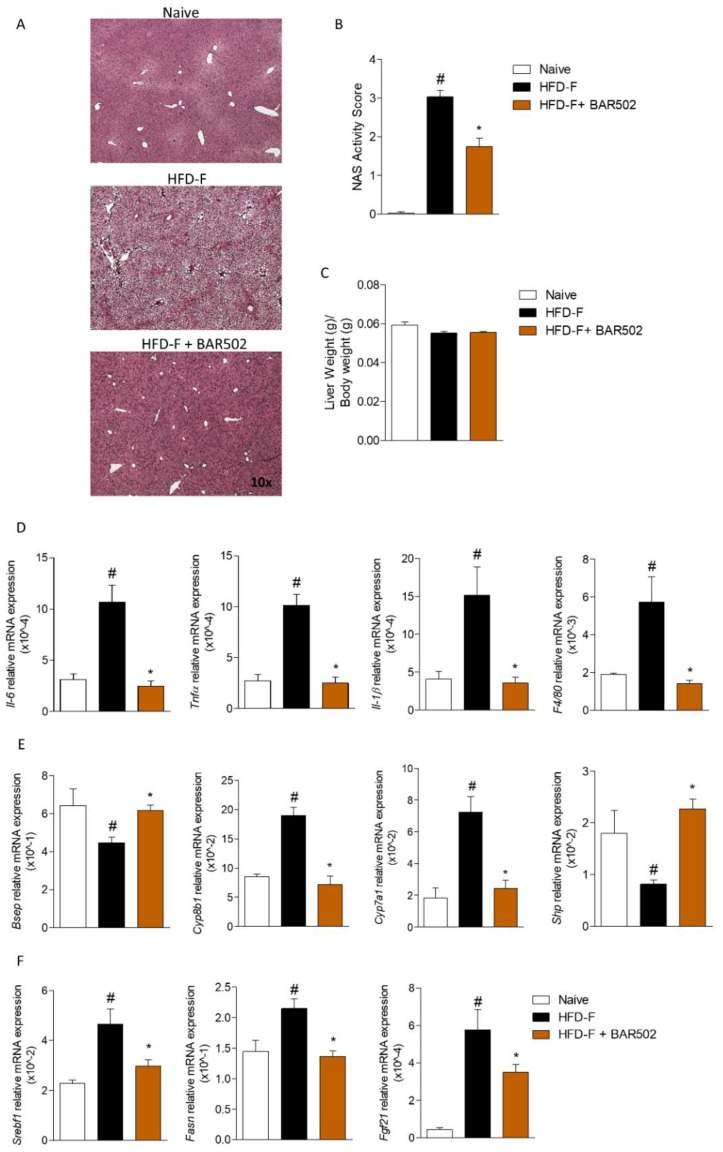
BAR502 attenuated non-alcoholic steatohepatitis (NASH) like features and redirect lipid partition in mice fed HFD–F. BAR502, 20 mg/kg/day, was administered by gavage in C57BL/6J male mice starting on day 10 of high fat diet (HFD) and fructose for additional 11 weeks. (**A**) Hematoxylin and eosin (H&E) staining on mice liver tissues showing severe steatosis and ballooning of hepatocytes in mice feed a HFD–F for 12 weeks, magnification 10×; (**B**) NAS activity score assessed in at least 5 different fields per liver in a blind manner; (**C**) Liver weight expressed as ratio Liver weight to Body weight. Panels **D**–**F**: Analysis of hepatic gene expression conducted with RT-PCR. Liver expression of (**D**) markers of inflammation (Il-6, Tnfα, Il-1β, F4/80), (**E**) genes involved in bile acids metabolism, (**F**) genes involved in lipid synthesis. The RT-PCR values were normalized against β2-Microglobulin and Gapdh, and the relative mRNA is expressed according the Ct method as described in Materials and Methods. The data shown are mean ± SEM of 7–10 mice/group. In each panel # denotes *p* < 0.05 versus Naïve mice, * denotes *p* < 0.05 versus HFD–F mice.

Mice feed a HFD–F for 12 weeks developed NASH-like features as revealed by H&E staining of liver sections, with micro-vesicular steatosis, hepatocytes ballooning, and lobular inflammation and inflow of macrophages (Figure 2A) which resulted in a significant increase in the liver steatosis score and liver inflammatory score (NAS activity score) (Figure 2B, # *p* < 0.05 versus naïve mice, * *p* < 0.05 versus HFD–F mice). Fittingly, mice on HFD–F had an increased expression of pro-inflammatory genes (Il-6, Tnf-α, Il-1β) and F4/80, a marker for pro-inflammatory macrophages (Figure 2D, # *p* < 0.05 versus naïve mice, * *p* < 0.05 versus HFD–F mice). No effects were observed on liver weight expressed as ratio of Liver weight/Body weight (Figure 2C). Treating mice with BAR502 improved liver histopathology by reducing both steatosis and ballooning and inflammation (Figure 2B), and reversed the upregulation of pro-inflammatory genes caused by HFD–F (Figure 2D, # *p* < 0.05 versus naïve mice, * *p* < 0.05 versus HFD–F mice).

As shown in Figure 2E, feeding a HFD–F significantly up-regulated the expression of Srebf1 (Srepb1c) and Fasn along with Cyp7a1 and Cyp8b1, but down-regulated the expression of Bsep, thus modulating the bile acids synthesis and transport across hepatocytes (Figure 2E,F, # *p* < 0.05 versus naïve mice, * *p* < 0.05 versus HFD–F mice) as well as expression of Fxr, Shp and Fgf21 (Figure 2G, # *p* < 0.05 versus naïve mice, * *p* < 0.05 versus HFD–F mice). Again, treating mice with BAR502 reversed this pattern and restored FXR signaling in the liver (Figure 2, # *p* < 0.05 versus naïve mice, * *p* < 0.05 versus HFD–F mice).

### 3.3. BAR502 Ameliorates Liver Fibrosis Caused by HFD–F

**Figure 3:**
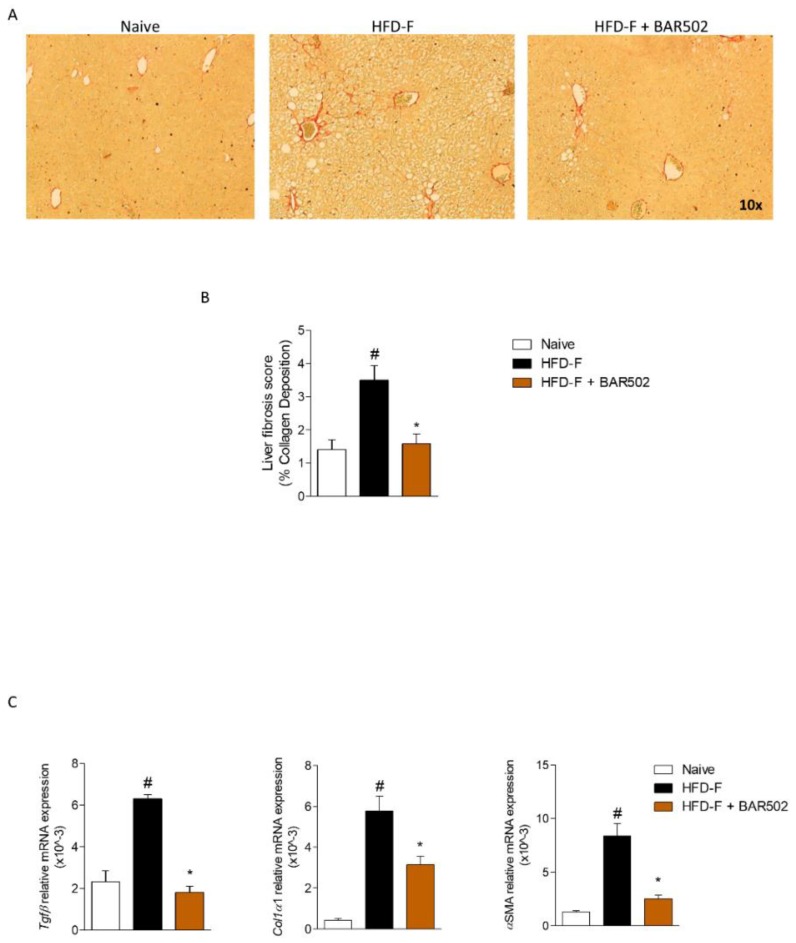
BAR502 ameliorated Fibrosis caused by HFD–F administration. BAR502, 20 mg/kg/day, was administered by gavage in C57BL/6J male mice starting on day 10 of HFD and fructose for an additional 11 weeks. (**A**) Sirius Red staining of liver sections. The images shown are representative of at least 10 others, each one obtained from an individual mouse, showing a similar pattern of regulation. Magnification 10×. (**B**) % of Collagen Deposition (Liver Fibrosis Score) was measured as described in Material and Methods by Image J analysis. (**C**) Hepatic expression of pro-fibrotic genes Tgfβ, Col1α1, and αSma. The RT-PCR values were normalized against β2-Microglobulin and Gapdh, and the relative mRNA is expressed according the Ct method as described in Materials and Methods. The data shown are mean ± SEM of 7–10 mice/group. In each panel # denotes *p* < 0.05 versus Naïve mice, * denotes *p* < 0.05 versus HFD–F mice.

Scoring of Sirius red stained liver sections demonstrated that feeding mice a HFD–F increases the liver collagen deposition, resulting in a moderate, but significant, fibrosis (Figure 3A,B, # *p* < 0.05 versus naïve mice, * *p* < 0.05 versus HFD–F mice). These histopathology findings were confirmed by the analysis of expression of canonical fibrosis markers Tgfβ, Col1α1, and αSma (Figure 3C, # *p* < 0.05 versus naïve mice, * *p* < 0.05 versus HFD–F mice) [17]. As shown in Figure 3, the collagen deposition was significantly attenuated by treating HFD–F mice with BAR502 that also reduced hepatic expression of Tgfβ, Col1α1, and αSma mRNAs (Figure 3C, # *p* < 0.05 versus naïve mice, * *p* < 0.05 versus HFD–F mice).

### 3.4. RNA Seq Analysis

**Figure 4:**
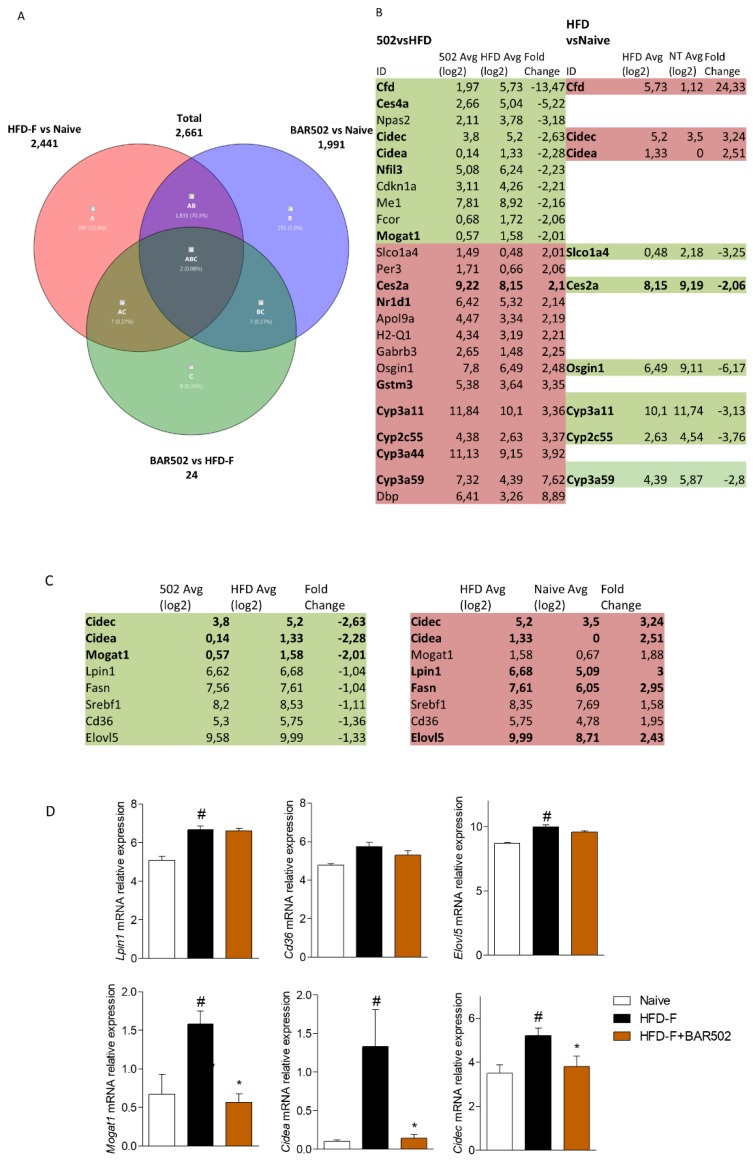
RNA-seq of BAR502 treated mice. BAR502, 20 mg/kg/day, was administered by gavage in C57BL/6J male mice starting on day 10 of high fat diet (HFD) and fructose for an additional 11 weeks. (**A**) Venn diagram of differentially expressed genes showing the overlapping regions (identified as ABC, AC, AB, and BC sets) between the three experimental groups of mice (Fold Change <−2 or >2, *p* value < 0.05). (**B**) Table showing the fold change of expression of genes included in region C, AC, and ABC of the corresponding Venn Diagram. Panels **C**,**D**. Analysis of genes involved in triglycerides synthesis and lipid storage into the hepatocytes, that were found differentially expressed both in HFD–F mice compared to Naïve mice, both in HFD–F + BAR502 and HFD–F + UDCA compared to HFD–F mice. In each panel # denotes *p* < 0.05 versus Naïve mice, * denotes *p* < 0.05 versus HFD–F mice.

To further elucidate the effect of the dual FXR/GPBAR1 agonist on transcriptome regulation, total RNA extracted from the livers of each group of mice were subjected to RNAseq analysis. As illustrated in Figure 4, 12-weeks HFD–F modulated a large set of genes: up to 2441 gene transcripts resulted differentially expressed by the liver of HFD–F treated mice compared to naïve mice (Fold change ≤−2 and ≥+2). As shown by Venn Diagram’s analysis, BAR502 only slightly modulated this pattern, resulting in 24 differentially expressed transcripts in comparison to mice feed the HFD–F alone (Figure 4A). Further analysis revealed that among these 24 transcripts, nine genes were differentially modulated also by HFD–F in comparison with naïve mice. Specifically, the table shown in Figure 4B, demonstrated that three transcripts (Cfd, Cidec and Cidea) that were up-regulated in HFD–F mice compared to naïve mice were down-regulated by BAR502. Cidea and Cidec, also known as FSP27 [30,31,32,33], are not detectable in the normal liver but become highly expressed in response to fat deposition (Figure 4). In addition, as shown is Figure 4C,D, feeding a HFD–F increased uptake of FFA by hepatocytes resulting in the up-regulation of genes involved in synthesis of triacylglycerols including Sterol regulatory element-binding transcription factor 1 (Srebf1 also named Srebp1c), Fatty Acid Synthase (Fasn), Fatty Acid Elongase 5 (Elovl5), Monoacylglycerol O-Acyltransferase 1 (Mogat1), and Lipin 1. Conversely, six transcripts, that were down-regulated by HFD–F mice in comparison to naïve mice, were up-regulated by BAR502 treatment. Furthermore, up to 1999 genes resulted differentially expressed by BAR502 treated mice compared to naïve mice, demonstrating that BAR502 does not restore the baseline setting (Figure 4A). 

### 3.5. Effects of BAR502 on Adipose Tissues and Terminal Ileum

**Figure 5:**
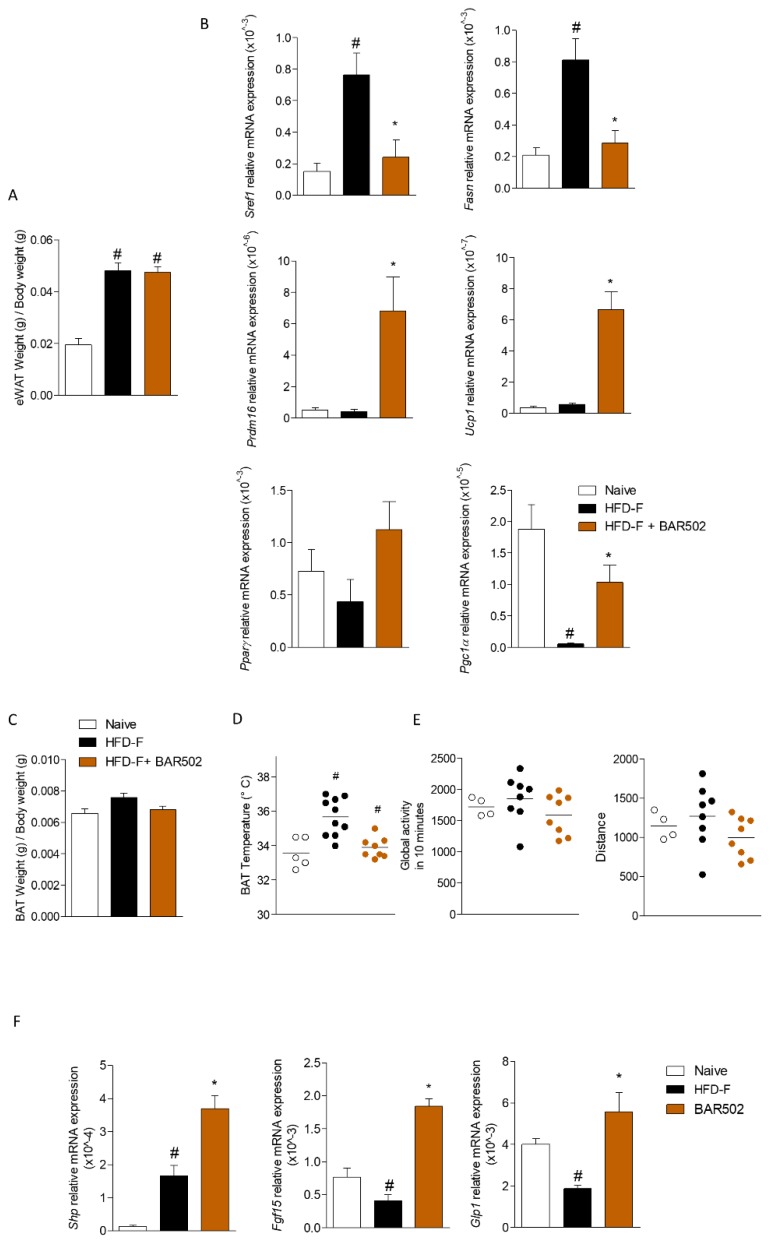
Effects of HFD–F on adipose tissue and ileum. BAR502, 20 mg/kg/day, was administered by gavage in C57BL/6J male mice starting on day 10 of HFD and fructose for additional 11 weeks. Panels **A**,**B**. Analysis of epididymal White Adipose Tissue (eWAT). (**A**) Ratio of eWAT weight to body weight. (**B**) Changes in mRNA expression of genes involved in: lipid synthesis and metabolism (Srebf1, Fasn), brite/beige trans-differentiation and β-oxidation (Prdm16, Ucp1, Pparγ, Pgc1α). Panels **C**–**E**. Analysis of Brown Adipose Tissue (BAT). (**C**) Ratio of BAT weight to body weight. (**D**) Thermogenic activity of BAT as measured by infrared spectroscopy. (**E**) Physical activity of mice expressed as Global activity and distance. The data showed in Panels **D**–**E** are recorded after 10 weeks of HFD–F. (**F**) Change in transcript levels of genes involved in regulation of intestinal metabolism (Shp, Fgf15, Glp1). The RT-PCR values were normalized against β2-Microglobulin and Gapdh, and the relative mRNA is expressed according the Ct method as described in Materials and Methods. The data shown are mean ± SEM of 7–10 mice/group. In each panel # denotes *p* < 0.05 versus Naïve mice, * denotes *p* < 0.05 versus HFD–F mice.

As shown in Figure 5, exposure to the HFD–F, significantly increased the ratio of epididymal white fat (eWAT) weight/body weight (Figure 5A, # *p* < 0.05 versus naïve mice). Feeding mice a HFD–F resulted in increased expression of lipidogenic pathway in epWAT as demonstrated by the increased expression of Srepb1c, Fasn, along with reduced expression of Pparγ and Pgc1α (Figure 5B, # *p* < 0.05 versus naïve mice, * *p* < 0.05 versus HFD–F mice). Feeding HFD–F slightly increased the Brown fat (BAT) weight/body weight and also increased the its basal thermogenic activity as measured by infrared spectroscopy analysis (Figure 5C,D). Treating mice with BAR502 reversed this pattern, and resulted in a robust induction of Prdm16, Ucp1, and Pgc1α (Figure 5B), thus promoting energy expenditure. No significant changes were recorded in terms of mice motor activity (Figure 5E). Treating mice with BAR502, reduced the basal thermogenic activity in comparison to mice fed a HFD–F alone (Figure 5D, * *p* < 0.05 versus HFD–F mice). 

Furthermore, the analysis of expression of FXR and GPBAR1 target genes in ileum revealed that HFD–F significantly reduced the expression of Fgf15 and Glp1 (Figure 5F, # *p* < 0.05 versus naïve mice). On the other hand, treating HFD–F mice with BAR502 increased the expression of Fgf15 and Shp (two FXR target genes), and Glp1 (a GPBAR1 target gene), confirming a FXR and GPBAR1 activation also at intestinal level (Figure 5F, # *p* < 0.05 versus naïve mice; * *p* < 0.05 versus HFD–F mice).

### 3.6. Effects of HFD–F on Bile Acid Synthesis and Excretion

**Figure 6:**
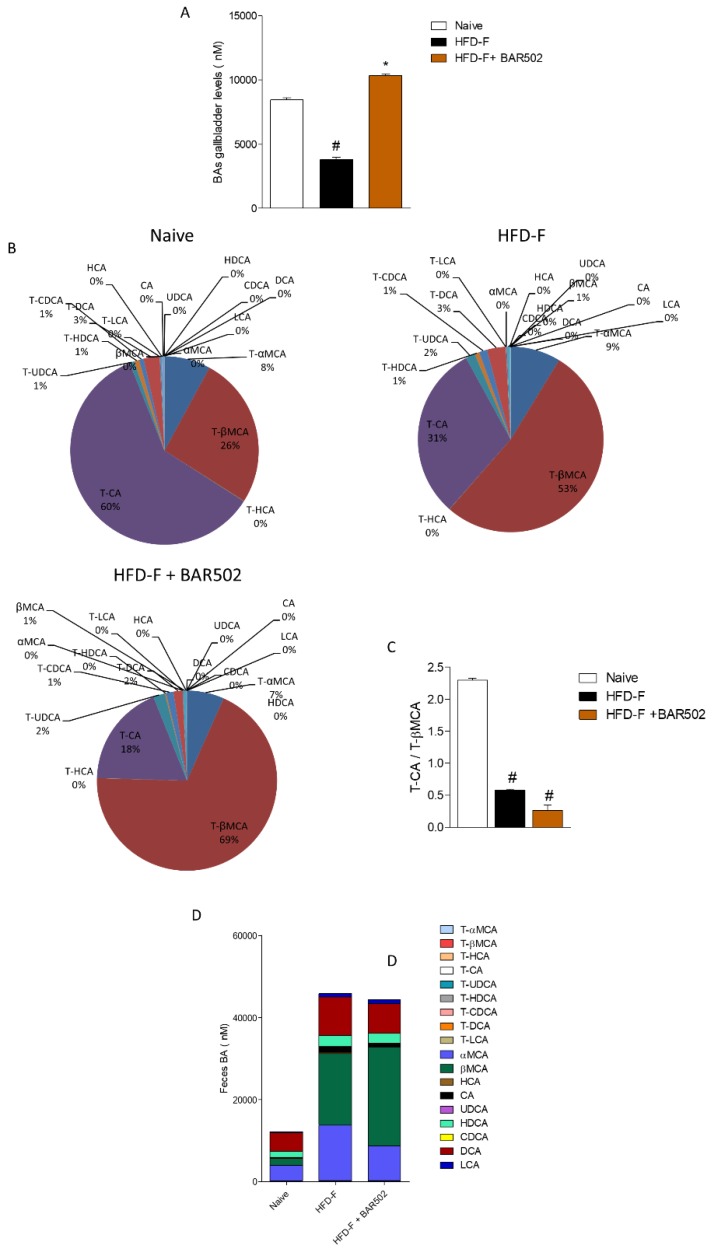
Analysis of gallbladder and fecal BAs composition. BAR502, 20 mg/kg/day, was administered by gavage in C57BL/6J male mice starting on day 10 of HFD and fructose for an additional 11 weeks. (**A**) Total concentrations of gallbladder bile acids. (**B**) Composition % of gallbladder bile acids pool in NT, HFD–F, and HFD–F + BAR502 mice. (**C**) Ratio tauro- cholic acid (T-CA)/tauro-beta muricholic acid (T-βMCA 0in gallbladder samples. (**D**) Composition of fecal bile acids pool. Data are the mean ± SEM of 7–10 mice per group. In each panel # denotes *p* < 0.05 versus Naïve mice, * denotes *p* < 0.05 versus HFD–F mice.

As shown in Figure 6A, mice fed a HFD–F were characterized by a profound remodeling of bile acid metabolism. In addition to the fact that total bile acids were reduced by approximately 60%, feeding mice with a HFD–F resulted in a marked reduction of CA and its taurine conjugate, T-CA (*p* < 0.05 vs. naïve mice). In contrast, the content of other primary bile acids such as T-βMCA remained relatively stable, thus resulting in a profound alteration of the T-CA/T-βMCA ratio (Figure 6B,C). Because T-CA is the predominant FXR agonist in mice, and T-βMCA functions as a FXR antagonists, this bile acid pattern might explain the effect of HFD–F on liver FXR signaling [37,38,39]. Treating mice with BAR502 did not reverse this pattern, howeverit increased the conversion of T-βMCA in βMCA by approximately 1.5-fold (*p* < 0.05 vs. HFD–F mice), thus reducing the FXR antagonism (Figure 6D). 

### 3.7. Analysis of Fecal Microbiota Composition

**Figure 7:**
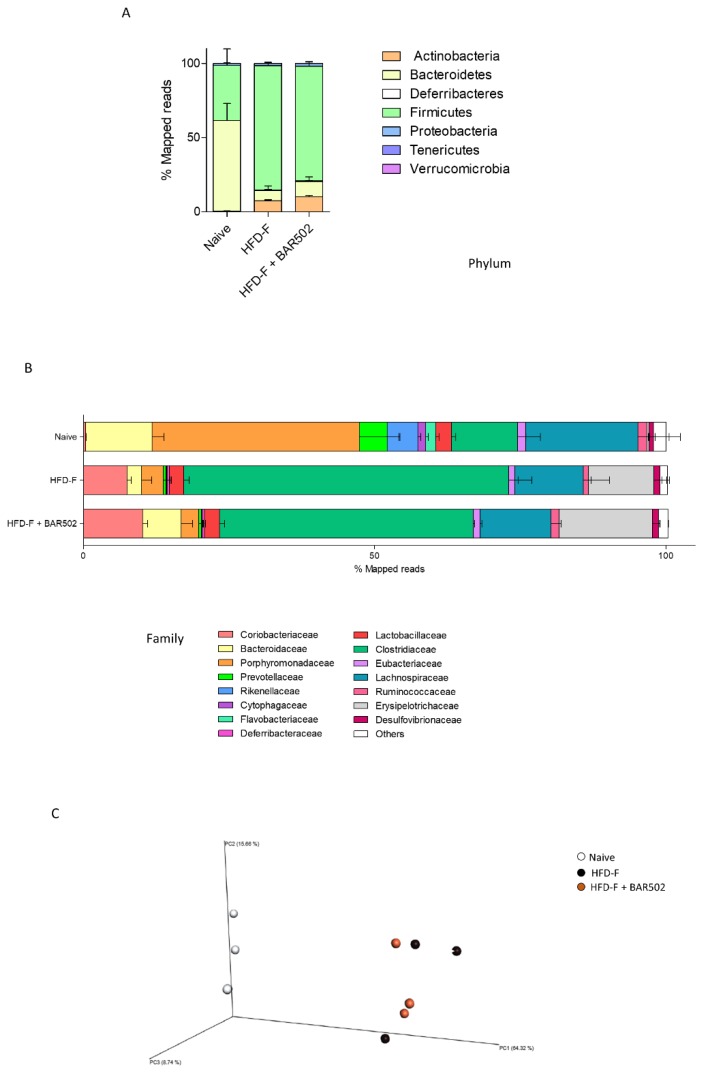
Analysis of fecal microbiota composition BAR502, 20 mg/kg/day, was administered by gavage in C57BL/6J male mice starting on day 10 of HFD and fructose for an additional 11 weeks. (**A**) Relative abundance of Phyla, calculated as percent of Mapped Reads. (**B**) Relative abundance of bacterial Families, calculated as percent of Mapped Reads. (**C**) Quantitative β analysis of PCoA conducted by family (Bray-Curtis analysis), that showed a major dissimilarity between untreated mice and mice under HFD–F.

To obtain further information about the impact of HFD–F and different treatments, we have analyzed stool samples, collected at the end of the experiment, for their microbial diversity. Metagenomics analysis revealed significant differences in microbiome composition expressed as relative abundance of Phyla, calculated as amount of Mapped Reads. As displayed in Figure 7A, the HFD–F treatment caused a severe dysbiosis reducing the amount of *Bacteroidetes* (*p* < 0.05 vs. naïve mice) and increasing the amount of *Firmicutes* and *Actinobacteria* (*p* < 0.05 vs. HFD–F). Further investigation of the microbiota structure by analysis of the relative abundance of bacterial Families, calculated as percent of Mapped Reads, revealed that the HFD–F administration reduced the relative amount of *Bacteroidaceae* and *Porphyromonadaceae*, and significantly increased the amount of *Coriobacteriaceae*, *Erysipelotrichaceae*, but especially of *Clostridiaceae*. These data were confirmed by quantitative β analysis of PCoA conducted by family (Bray-Curtis analysis), that showed a major dissimilarity between untreated mice and mice under HFD–F (Figure 7C). Treating mice with BAR502 increased the percent of *Bacteroidaceae* by approximately 3–4-fold, (*p* < 0.05 vs. HFD–F mice) and reduced the relative abundance of *Clostridiaceae* family by approximately 1.5-fold in comparison to mice feed HFD–F alone.

## 4. Discussion

In the present study we report that a dual FXR and GPBAR1 ligand, BAR502, exerts beneficial effects in a rodent model of NASH and fully reverses the development of NASH like features in mice feed an HFD–F, a well validated model of NASH and insulin resistance. Clinically, the beneficial effects exerted by BAR502 manifested by significant attenuation of body weight gain and increased BMI and Lee index caused by feeding mice with HFD–F. Because GPBAR1 agonism is thought to promote energy expenditure, these features are likely to reflect the benefit of simultaneous activation of this receptor. In addition, BAR502 effectively reduced liver fat accumulation, as measured by assessing the steatosis score, inflammation, as measured by assessing the inflammatory score as well as expression of inflammatory biomarkers such as Il-1β, Il-6, Tnfα, and F4/80, and severity of liver fibrosis, assessed by measuring the fibrosis score, as well as the expression of markers of extracellular matrix deposition including Col1α, αSma, and Tgfβ (Figure 2 and Figure 3).

Because GPABR1 is not expressed by liver parenchymal cells, we have examined whether and how BAR502 impacts on liver FXR signaling. A detailed analysis of the FXR pathway demonstrated that feeding mice with a HFD–F impairs FXR gene expression as well as liver FXR signaling [37]. Thus, mice feed a HFD–F had reduced expression of Bsep and Shp, two positively regulated FXR-target genes, along with increased expression of Cyp7a1 and Cyp8b1, two negatively regulated FXR-target genes. The mechanisms that support attenuation of liver FXR signaling in this model are likely several. From one hand, the HFD–F represents a potent inflammatory driver in the liver, as demonstrated by the 10–15-fold induction in the expression of pro-inflammatory mediators such as Tnfα. Of relevance it is well established that FXR signaling is blunted in inflammatory settings [38]. Additionally, analysis of gallbladder bile acids demonstrated that HFD–F resets bile acid synthetic pathways, reducing CA and T-CA, which was not compensated for by changes in other FXR agonistic bile acid species. Indeed, mice feed an HFD–F were characterized by a robust increase in the relative amount of T-βMCA, a relatively potent FXR antagonist [39,40]. Thus, changes in T-CA/T-βMCA ratio are likely to be the underlying mechanism that support the compromised FXR signaling observed in this model. In contrast, feeding mice with BAR502 effectively rescued FXR signaling as shown by the robust resetting of canonical FXR-target genes, including the upregulation of Shp and Bsep and the suppression of Cyp7a1 and Cyp8b1.

**Figure 8:**
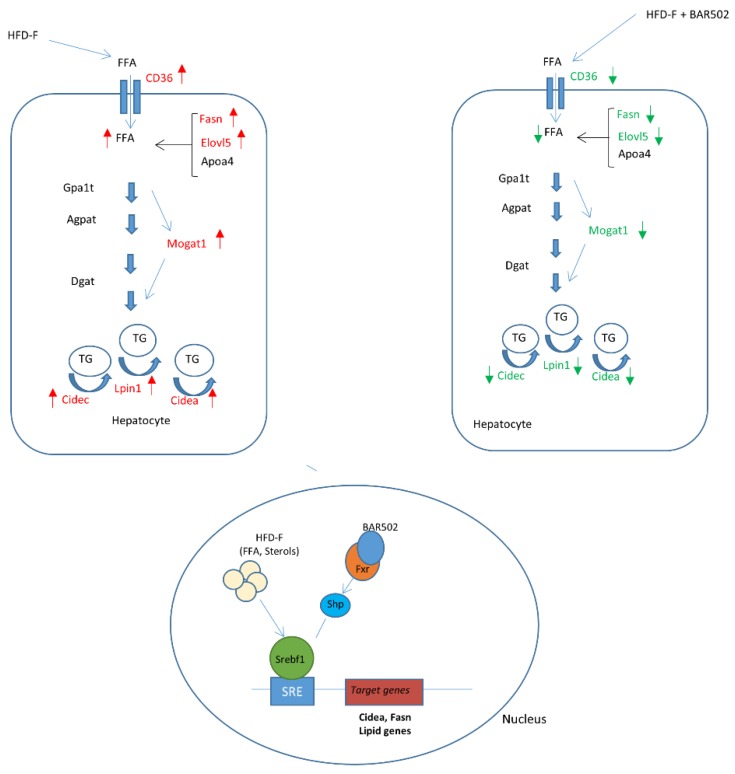
Schematic representation of the transcriptional regulation and role of CIDE pathway in controlling hepatic lipid metabolism (red and green arrows indicate respectively up- and down- regulated genes) Under HFD–F treatment the gene expression of protein involved in triglycerides synthesis and storage, resulted increased. Particularly the HFD–F via Srebf1 activation, promoted the transcription of lipidogenic genes, such as Fasn and Cidea. BAR502 activates FXR thus suppressing a cluster of genes downstream to Srebf1 including fatty acid synthase (Fasn) and Cell death-inducing DFF45-like effector (CIDE) genes, Cidea and Cidec, involved in lipid droplets formation and triglycerides storage.

Because BAR502 effectively attenuated the development of NASH like features in the liver, we have carried out a detailed transcriptome. While, in general, present results are consistent with a previous report (29), the RNA seq analysis shown here allowed the identification of novel therapeutic targets for the dual FXR/GPBAR1 ligand. Indeed, while exposing mice to the HFD–F resulted in wide modification of liver transcriptome architecture with up to 2441 differentially regulated transcripts in comparison to mice fed a regular diet, strongly highlighting the plasticity of mammalian metabolism in adapting to diet changes. Despite the fact that BAR502 effectively reversed development of NASH-like features, only a surprisingly low number of transcripts were modulated by this agent, i.e., 24 transcripts. The pathway analysis shown in Figure 4, however, revealed that effects exerted by BAR502 were clustered in a string of genes: Cd36, Elov5, Srebf1, Fasn, Lpin1, Mogat and Cidea and Cidec, critically involved in lipid metabolism and lipid droplet formation and growth in hepatocytes. These targets were further confirmed by RT-PCR analysis. The family of CIDE proteins includes three members in mice (Cidea, Cideb, and Cide c/FSP27) and humans (CIDEA, CIDEB, and CIDEC), that are encoded by set of genes that share a common N-terminal CIDE-N domain and a C-terminal CIDE-C domain [30,31,32,33]. Despite the fact that a significant homology has been detected between the CIDE-N domain of CIDE proteins and the regulatory domains of the apoptotic DNA fragmentation factors, caspase-activated nuclease DFF40 and DFF45 (DFF40 inhibitor), CIDE proteins are essentially lipid droplets (LD)-associated protein in mouse adipocytes. Importantly, genetic studies have shown that CIDE protein are essential for regulation of LD traffic in adipocytes [40,41,42,43,44,45,46,47,48]. Indeed, both Cidea and Cidec localize on the surface of LD and are particularly enriched at contact sites of LD to promote their fusion and growth by lipid exchange and transfer [40,41,42,43,44,45,46,47,48]. Several factors are involved in Cidea/Cidec-mediated LD fusion, including Plin1, Rab8a, MSS4, and AS160. Like perlipins, expression of Cidea and Cidec is regulated by peroxisome proliferator-activated receptor γ (PPARγ), and liver-x-receptor (LXR) and treatment of lean or obese mice with the PPARγ or LXR agonists up-regulates Cidea and Cidec expression in white adipose tissue increasing lipid deposition [40,41,42,43,44,45,46,47,48,49]. In addition, while Cidea and Cidec are predominantly expressed in adipocytes, and are poorly represented in the normal liver, their expression increases dramatically in the human steatotic liver [40] and a single nucleotide polymorphism of a G to T transversion in CIDEA exon 4, which is equivalent to a V115F substitution, is associated with higher body mass index in Swedish obese patients [50,51,52], and obesity and metabolic syndrome in Japanese and Chinese populations [50,51,52]. Controlling the correct amount of CIDE proteins is therefore important in maintaining lipid homeostasis in the liver, since lower levels of expression of CIDE proteins associates with reduced LD sizes and lower lipid storage capacity. In the presence of lipid-rich medium, CIDE proteins localized to LDs promote LD fusion and lipid storage in hepatocytes resulting in liver steatosis, suggesting a major role for Cidea and Cidec in regulating hepatic lipid storage under chronic high caloric intake. The mechanisms of regulation of Cidea and Cidec in the liver are still only partially elucidated. While Ppar-α and -γ increases the transcription of Cidea and Cidec in adipocytes, Srebp1c has been identified as the main regulator of the expression of Cidea in primary hepatocytes. In the liver, Srebp1c mediates the induction of Cidea gene expression caused by insulin [40,41,42,43,44,45,46,47,48,49]. Furthermore, overexpression of Cidea in hepatocytes promotes lipid droplets formation, whereas knocking down of Cidea expression partially reverses the effect of Srebp-1c on lipid accumulation. In obese mice and humans, the up-regulation of Srebp1c expression is considered a major driver for excessive lipogenesis and insulin resistance [41]. This finding suggests that Cidea mediates downstream effects of Srebp-1c pathway causing lipid droplet accumulation and fusion in hepatocytes and the development of liver steatosis in conditions of insulin resistance and obesity. Similarly, to Cidea and Cidec, also the Fasn is tightly regulated by SREBP1 protein. Since Srebp1c is negatively regulated by FXR, and BAR502 reduced the liver expression of Srebp1c, as shown in the schematic representation in Figure 8, we speculated that negative regulation of Cidea and C in mice treated with BAR502 s mediated, at least in part, through negative regulation of Srepb1c [53,54,55,56,57].

In addition to these findings we have examined the effects of BAR502 on the structure of the intestinal microbiota in mice feed a HFD–F. The main change observed in mice feed a HFD–F was a dramatic increase in the amount of intestinal *Firmicutes* in comparison to naïve mice fed a standard chow diet [58]. These findings are highly consistent with the pattern observed in obese humans [59,60]. Since *Firmicutes* have an improved energy yield from intestinal contents (e.g., short-chain fatty acids including acetate, butyrate, and propionate), they are thought to accelerate obesity and NAFLD, mostly likely through *de novo* lipogenesis thus establishing a potential role for gut microbiota in human NAFLD. However, since the pathogenesis of obesity is multifactorial, other studies have failed to confirm this tight correlation [61], and other have shown that exposure of mice to an HFD drives obesity regardless the composition of the intestinal microbiota [62]. Importantly, treating mice with BAR502 increased the relative amount of *Bacteroidaceae* by approximately 3–4-fold in comparison to mice fed HFD–F alone. Since this family has been shown to be reduced in NASH and its expansion associates with an improvement in lipid metabolism, we speculated that part of the beneficial effects exerted by BAR502 could be mediated by its activity toward the intestinal microbiota. Furthermore, it has been shown that an activation of intestinal FXR signaling, reshapes the microbiota toward the production of bile acids that activate a GPBAR1/Glp1 axis, improving the hepatic insulin sensitivity and leading to an increase in white adipose tissue browning [63,64]. These findings are consistent with the induction of expression of Shp, Fgf15, and Glp1 we have documented in the terminal ileum, despite the increased levels of the FXR antagonist T-βMCA. This result could be explained by an increased conversion of T-βMCA in βMCA by the microbiota families with *bile salt hydrolase* enzymatic activity, such as *Bacteroidetes* [65].

The present study has several limitations. First of all, since control mice fed a HFD–F diet were not gavaged with vehicle, it cannot be excluded that some of the effects exerted by BAR502 could be linked to the stress caused by the gavage. Additionally, this is an animal study and its translation to human settings needs to be proven in clinical trials.

In summary, we have shown that BAR502, a dual FXR/GPBAR1 ligand, reverses steatohepatitis and fibrosis caused by chronic exposure of mice to a high caloric diet. By transcriptome analysis we have revealed that activation of FXR and GBAR1 in the liver, adipose tissues and intestine, lead to a regulation of lipid biosynthesis and inhibition of genes involved in fatty acid deposition. These results are of pharmacological interest and support the progression of BAR502 into clinical trials in NASH.

## Abbreviations

NAFLD, non-alcoholic fatty liver disease; NASH, non-alcoholic steatohepatitis; BARs, bile acid activated receptors; FXR, farnesoid X receptor; GPBAR1, G-protein coupled bile acid receptor; TGR5, Takeda-G-protein receptor 5; HFD–F, high fat diet and fructose; CA, cholic acid; CDCA, chenodeoxycholic acid; OCA, obeticholic acid; UDCA, ursodeoxycholic acid; αMCA, alpha muricholic acid; βMCA, beta muricholic acid; LCA, litocholic acid; AC, abdominal circumference; AUC, area under curve; BMI, body mass index; AST, aspartate transaminase; ALT, alanine transaminase; OGTT, oral glucose tolerance test; T-CA, tauro- cholic acid; T-CDCA, tauro-chenodeoxycholic acid; T-αMCA, tauro- alpha muricholic acid; T-βMCA, tauro-beta muricholic acid; T-UDCA, tauro- ursodeoxycholic acid; T-LCA, tauro-litocholic acid; eWAT, epididymal white adipose tissue; BAT, brown adipose tissue; HOMA, Homeostatic Model Assessment.
